# Association between cardiac rehabilitation and LDL-levels, adherence to guideline-recommended medication and mortality rate after myocardial infarction

**DOI:** 10.1016/j.ijcrp.2025.200444

**Published:** 2025-06-07

**Authors:** Ahmad Agam, David Vadsholt, Kristian Kragholm, Lauge Klement Moltke Østergaard, Peter Bisgaard Stæhr, Gitte Nielsen, Henrik Vadmann

**Affiliations:** a Department of Cardiology, Aalborg University Hospital, Aalborg, Denmark; b Department of Cardiology and Endocrinology, North Denmark Regional Hospital, Hjoerring, Denmark; c Aalborg University Hospital, Denmark; d Department of Cardiology, Rigshospitalet, Copenhagen University Hospital, Copenhagen, Denmark

**Keywords:** Cardiac rehabilitation, Post-myocardial infarction, Secondary prevention, Revascularization

## Abstract

**Aim:**

This study aims to identify three-month and one year mortality rate, LDL level and adherence to guideline-recommended medication in patients with myocardial infarct (MI) receiving cardiac rehabilitation (CR) compared to patients who do not.

**Method:**

In this retrospective study, patients hospitalized in North Denmark Regional Hospital in Hjoerring (capture population 200.000) with acute coronary syndrome between January 1st, 2017, to December 31st, 2021, were included. Baseline characteristics, initial treatment of revascularization and all-cause mortality were examined through the Danish National Patient Registry, the Regional Cardiac Rehabilitation Database, and medical chart review. Patients were grouped by revascularization (yes/no) during hospitalization and CR. Adjusted Cox proportional regression model was used to assess differences in mortality and LDL levels.

**Results:**

A total of 1209 myocardial infarction (MI) survivors were included in this study. A total of 1209 myocardial infarction (MI) survivors were included. Significant LDL reductions at 6- and 12-month follow-ups were observed in patients receiving both cardiac rehabilitation (CR) and lipid-modifying therapy at baseline (p = .001), but not in those without CR. In revascularized patients, use of multiple antithrombotic agents was lower in the no CR group at three months (57.1 % vs 78.8 %, p = .002) and one year (60 % vs 78.5 %, p = .010). Three-month mortality rate was higher among patients who did not undergo CR, both in the revascularization group (19 % vs 2 %, p = 0.001) and the non-revascularization group (18 % vs 3 %, p = 0.001).

**Conclusion:**

Patients undergoing CR were associated with lower LDL-levels, higher adherence to guideline-recommended medication and lower mortality rate at three-month follow-up.

## Highlights

• A total of 1,209 MI survivors included; Cardiac Rehabilitation (CR) participation rate was high at 95.6 %.
• LDL fell at 6 and 12 months in patients receving CR plus lipid therapy (p = 0.001), but not in those without CR.
• Revascularized no-CR patients had less antithrombotic use at 3m (57 % vs 79 %) and 1y (60 % vs 79 %).
• Three-month mortality was higher in patients without CR: 19 % vs 2 % (rev) and 18 % vs 3 % (non-rev), both p = 0.001.

## 1 Introduction

Coronary artery disease (CAD) is a leading cause of death worldwide, both in high-as well as middle-to-low-income countries [1]. It is projected to be the leading cause of death worldwide by 2030 [2]. The increasing prevalence of CAD is due to the increase in many of the risk factors such as type 2 diabetes mellitus, obesity, hypertension, and ageing of the population [3]. Therefore, a relevant intervention such as cardiac rehabilitation (CR) is valuable to improve the wellbeing and health-related quality of life of patients with heart disease and decrease risk factors and prevent reoccurrence of cardiovascular events. CR is a complex intervention that seeks to improve the functional capacity, and wellbeing of patients with heart disease, it is offered to patients who have been hospitalized with acute coronary syndrome (ACS) due to the documented improvement in symptoms and reduction of mortality rate ([[3], [4], [5], [6]]).

CR significantly reduces secondary cardiovascular events and mortality and is a class 1A recommendation by European Society of Cardiology (ESC) [7]. Several studies have found that receiving CR after atherosclerotic cardiovascular disease events or revascularization reduce CV hospitalizations, ACS, CV mortality and, in some studies, all-cause mortality [[8], [9], [10]]. Although CR is recommended, it remains an underutilized intervention, and many eligible patients fail to enroll or complete CR programs [11]. However, it is important to note that the benefits of CR are variable across different cardiovascular conditions with the strongest mortality and morbidity benefits after acute myocardial infarction (AMI) and coronary artery bypass graft surgery [11].

Despite the documented improvement of CR and the recommendations in American and European guidelines the participation is still not optimal, and we lack an understand of how many of those are potential candidates for CR. [[12], [13], [14]]. CR is not standardized with varying degrees of quality and program length but mainly consists of three phases. The acute treatment until discharge, the time from discharge to independent living and lastly the follow-up ([3,15]). Knowing the benefits of CR, it emphasizes the importance of a future high level of attendance and quality of CR.

**Aims:** The aims of this study were to identify three-month and one year mortality rate, LDL level and adherence to guideline-recommended medication in patients with MI receiving CR compared to patients who do not receive CR.

## 2 Methods

This is a retrospective study conducted at the department of cardiology, North Denmark Regional Hospital in Hjoerring (capture population 200.000). The study population consist of patients hospitalized in North Denmark Regional Hospital with a diagnosis of MI between January 1sts 2017 to December 31st, 2021.

### 2.1 Study population

The study population was identified through the Danish National Patient Registry (DNPR) and Regional Cardiac Rehabilitation Database (RCRD). The DNPR contains information on hospital admission and discharge dates, diagnosis, mortality, and procedures [8]. The RCRD contains information on participation in CR. Both databases were utilized to categorize patients according to post-MI CR status. The positive predictive value of myocardial infarction (ICD-10 codes: I219) in the DNPR has been found at 97 % previously [16]. Patients not registered in the RCRD were manually assessed through medical chart review. The National Prescription Registry was used to identify redeemed prescriptions for antithrombotic and lipid modifying medications after MI. The Danish registries are of high quality and previously described in detail [[17], [18], [19]].

Patients with type 2 myocardial infarction were included and were defined by a rise and fall of cardiac biomarkers without any evidence of stenosis. Details of the CR program in Denmark are provided in Table A in the supplementary material (20).

### 2.2 Exclusion

The exclusion criteria for this study are as follows: Patients who died during hospitalization were excluded from the analysis. Additionally, patients obtained solely from the RCRD with missing data on mortality and medication were excluded when evaluating adherence to medication. The period for examining medical adherence was from myocardial infarction (MI) discharge up to 3 months of follow-up, and only those patients surviving up to 3 months post-MI were included in the analysis. Lastly, patients diagnosed with unstable angina pectoris (UAP) were not included in this study.

Exposure to CR was identified by (a) the patient was registered in the RCRD with MI and underwent CR, (b) they had a referral to community CR or (c) underwent CR in outpatient clinics at the hospital, according to medical records.

### 2.3 Outcomes

The outcome of the study aimed to assess the quality of CR in patients diagnosed with myocardial MI. This included evaluating the proportion of CR participation, initial treatments such as revascularization and guideline-recommended therapies (aspirin, P2Y12 inhibitor, lipid-modifying drugs including statins medications). All-cause mortality rates were examined at three months and one year follow-up, with survival probability starting at 100 % at 90 days. Medical adherence was assessed through redeemed prescriptions: 1) from MI discharge up to 90 days post-discharge, and 2) from 9 months to 12 months post-MI discharge. LDL levels were analyzed at baseline, 6-month, and 12-month follow-ups for patients using lipid-modifying drugs including statins, comparing those who received CR to those who did not. Baseline LDL levels were analyzed for patients receiving lipid-modifying drugs including statins at the start of the study, as well as at the 6-month and 12-month follow-up periods. Lipid-modifying drugs including statins included statins, ezetimibe, icosapent ethyl, and PCSK9 inhibitors.

Additionally, the proportion of patients achieving LDL targets after ESC/EAS guidelines 2016 (1.7 mmol/L) [20] and 2019 (1.4 mmol/L) [21] at 6 and 12 months follow-up stratified by CR status and baseline statins or other lipid-modifying therapy were also assessed.

### 2.4 Covariates

Covariates consist of age, sex, smoking status, weekly alcohol consumption, living alone, connection to the labor market, type of MI, body mass index (BMI), frailty at discharge, coronary angiography (CAG), antidiabetic medication, intake of lipid-modifying drugs including statins and familial predisposition to ischemic heart disease defined as first-degree relative with debut of MI before 65 years for women and 55 years for men.

### 2.5 Statistical analysis

Continuous data was expressed as means with standard deviation. For categorical variables, frequency and relative frequency defined by the percentages of the total number of individuals were used. To examine the differences between continuous data a paired sampled *t*-test was utilized. For categorical variables, the Chi-square test was used. The association between CR and mortality was analyzed with Kaplan-Meier estimator and plotted as well as Cox Proportional Hazard Regression Model. Hazard ratio (HR) was expressed with 95 % confidential interval (CI). P-values <0.05 were considered significant for rejecting the null hypothesis. All statistical analyses were conducted using SAS, version 9.4 (NC, Cary, USA).

### 2.6 Ethics

In Denmark patient consent is not required for retrospective register-based studies. The data responsible unit in the North Denmark Region has approved the project (ID 2018-169).

## 3 Results

### 3.1 Study population

**Fig. 1:**
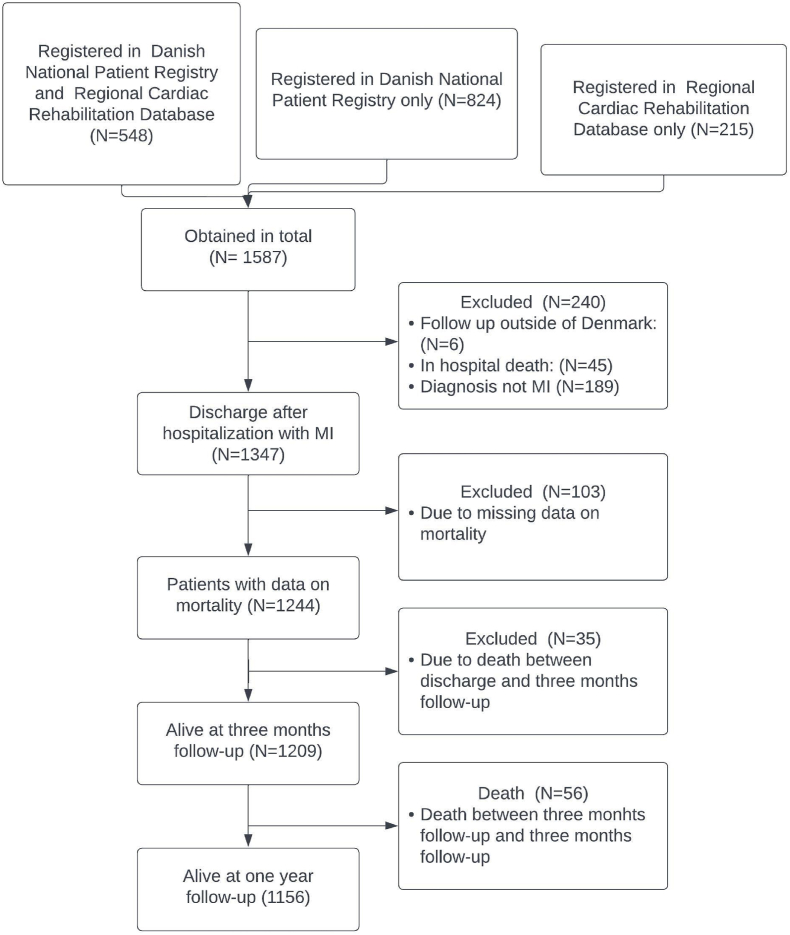
The identified study population and excluded patients.

Patients were grouped based on whether they had undergone revascularization during hospitalization and if CR was received. The final study sample consisted of 1209 MI survivors alive at three months follow-up. Fig. 1 illustrates the inclusion and exclusion process. A total of 965 underwent revascularization and 923 of these received CR thus resulting in 95.6 % participation. The rest did not undergo revascularization and only 155 of those received CR (63.5 %). A total of 56 patients died between the three month and one year follow-up, leaving 1156 patients alive for the one year follow-up analysis.

### 3.2 Baseline characteristics

Baseline characteristics of the study population from this study can be seen in Table 1. The mean age of the study population was 68 years. Overall, 12 % of the patients were classified as obese, while 18.9 % were overweight. Additionally, 15.4 % of all MI survivors were receiving antidiabetic treatment, and were receiving lipid drugs.

**Table 1:** Baseline characteristics at 90 days post-discharge for patients with and without revascularization during MI admission.

Baseline Characteristics at 90 days post-discharge	Total (n = 1209)	Revascularization (n = 965)		No revascularization (n = 244)	
		Cardiac rehabilitation		Cardiac rehabilitation	
		No (n = 42, 4.4 %)	Yes (n = 923, 95.6 %)	No (n = 89, 36.5 %)	Yes (n = 155, 63.5 %)
Mean age, (SD)	67.83 (12.67)	67.11 (12.11)	66.76 (11.82)	77.04 (14.05)	69.11 (14.59)
<65 years, n (%)	486 (40 %)	18 (42.9 %)	392 (42.5 %)	15 (16.9 %)	61 (39.4 %)
65–80 years, n (%)	516 (42.7 %)	20 (47.6 %)	408 (44.2 %)	32 (36.0 %)	56 (36.1 %)
>80 years, n (%)	207 (17.1 %)	4 (9.5 %)	123 (13.3 %)	42 (47.2 %)	38 (24.5 %)
Male, n (%)	815 (67.4 %)	36 (85.7 %)	651 (70.5 %)	41 (46.1 %)	87 (56.1 %)
Coronary angiography, n (%)	1108 (91.6 %)	42 (100 %)	923 (100 %)	45 (50.6 %)	102 (65.8 %)
Working	302 (25 %)	7 (16.7 %)	247 (26.8 %)	6 (6.7 %)	42 (27.1 %)
Retirement	545 (45.1 %)	17 (40.5 %)	396 (42.9 %)	67 (75.3 %)	65 (41.9 %)
Active/former smoker	425 (35.2 %)	26 (61.9 %)	273 (29.6 %)	53 (59.6 %)	73 (47.1 %)
Obese	145 (12 %)	8 (19 %)	99 (10.7 %)	15 (16.9 %)	23 (14.8 %)
Overweight	229 (18.9 %)	13 (31.0 %)	147 (15.9 %)	30 (33.7 %)	39 (25.2 %)
Antidiabetics, n (%)	183 (15.1 %)	6 (14.3 %)	143 (15.5 %)	16 (18.0 %)	18 (11.6 %)
Clinical frailty scale score above 4, n (%)	126 (10.4 %)	7 (16.7 %)	50 (5.4 %)	39 (43.8 %)	30 (19.4 %)
LVEF> 49 at discharge, n (%)	335 (27.7 %)	14(33.3 %)	246 (26.7 %)	26 (29.2 %)	49 (31.6 %)
Hypercholesterolemia	381 (31.5 %)	16 (4.2 %)	273 (71.7 %)	35 (9.2 %)	57 (15 %)

### 3.3 Medication adherence

#### 3.3.1 Three months follow-up

Table 2 presents medication adherence at the three-month follow-up, which included 1209 patients. Patients who underwent revascularization and participated in CR were more likely to receive multiple antithrombotic agents than those who did not participate in CR (78.8 % vs. 57.1 % p = .002). Additionally, 92.1 % of these patients received a lipid-modifying drugs including statins, compared to 76.5 % of those who did not participate in CR (p = .002).

**Table 2:** Medication at three months follow-up divided in patients undergoing revascularization and no revascularization and receiving cardiac rehabilitation and no cardiac rehabilitation. ∗∗P < 0.01 and ∗P < 0.05 were considered significant.

Medication at three months follow-up n = 1209	Revascularization			No revascularization		
	Cardiac rehabilitation			Cardiac rehabilitation		
	No (n = 42)	Yes (n = 923)	P- value	No (n = 89)	Yes (n = 155)	P- value
**Lipid-modifying drugs including statins**	32 (76.5 %)	850 (92.1 %)	.002∗∗	45 (50.6 %)	116 (74.8 %)	.0001∗∗
**Beta blocker**	22 (52.4 %)	528 (57.2 %)	.633	42 (47.2 %)	82 (52.9 %)	.426
**Renin-angiotensin-system inhibitor**	18 (42.9 %)	534 (57.9 %)	.058	32 (36 %)	74 (47.7 %)	.082∗∗
**Antidiabetics**	7 (16.7 %)	171 (18.5 %)	1.000	13 (14.6 %)	18 (11.6 %)	.551

**Loop diuretics**	8 (19 %)	153 (16.6 %)	.672	26 (29.2 %)	39 (25.2 %)	.548
**Spironolactone**	1 (2.4 %)	82 (8.9 %)	.252	9 (10.1 %)	19 (12.3 %)	.680
**Multiple antithrombotic agents**	24 (57.1 %)	727 (78.8 %)	.002∗∗	30 (33.7 %)	107 (69 %)	.0001∗∗
**No antithrombotic**	8 (19 %)	20 (2.2 %)	.0001∗	20 (22.5 %)	13 (8.4 %)	.0031∗∗

Among patients who did not undergo revascularization, those who participated in CR were also more likely to receive multiple antithrombotic agents than those who did not (69 % vs. 33.7 %, p = .0001). Similarly, this group was more frequently prescribed lipid-modifying drugs including statins compared to those who did not receive CR (74.8 % vs. 50.6 %, p = .0031).

#### 3.3.2 One year follow-up

Table 3 presents medication adherence at the one-year follow-up, which included 1156 patients. Patients who underwent revascularization and participated in CR were more likely to receive antithrombotic therapy after follow-up compared to those who did not participate in CR (78.5 % vs. 60 %, p = .010). This group was also more frequently prescribed lipid-modifying drugs including statins compared to those who did not receive CR (67.9 % vs 60 %, p = .304)

**Table 3:** Medication at one year follow-up divided into patients undergoing revascularization and no revascularization and receiving cardiac rehabilitation and no cardiac rehabilitation. ∗∗P < 0.01 and ∗P < 0.05 were considered significant.

Medication at one year follow-up n = 1156	Revascularization			No revascularization		
	Cardiac rehabilitation			Cardiac rehabilitation		
	No = (n = 40)	Yes (n = 899)	P- value	No (n = 76)	Yes (n = 141)	P- value
Lipid-modifying drugs including statins	24 (60 %)	610 (67.9 %)	.304	30 (39.5 %)	75 (53.2 %)	.064
Beta blocker	17 (42.5 %)	373 (41.5 %)	1.000	26 (34.2 %)	47 (33.3 %)	1.000
Renin-angiotensin-system inhibitor	15 (37.5 %)	477 (53.1 %)	.074	21 (27.6 %)	51 (36.2 %)	.229
Antidiabetics	6 (15 %)	151 (16.8 %)	1.000	10 (13.2 %)	11 (7.8 %)	.232
Loop diuretics	3 (7.5 %)	82 (9.1 %)	1.000	18 (23.7 %)	13 (9.2 %)	.006∗∗
Spironolactone	1 (2.5 %)	65 (7.2 %)	.354	6 (7.9 %)	14 (9.9 %)	.806
Antithrombotic after follow-up	24 (60 %)	706 (78.5 %)	.010∗∗	35 (46.1 %)	93 (66 %)	.005∗∗

Among patients who did not undergo revascularization, those who participated in CR were also more likely to receive antithrombotic therapy after follow-up compared to those who did not (66 % vs. 46.1 %, p = .005). Similarly, this group was more frequently prescribed lipid-modifying drugs including statins compared to those who did not receive CR (63.2 % vs. 39.5 %, p = .064).

### 3.4 LDL level

The paired samples tests for LDL levels were conducted at 6- and 12-months follow-up compared to baseline (Table 4). Significant differences were seen in both groups when receiving lipid-modifying drugs including statins at baseline and CR and those only receiving CR without lipid-modifying drugs at baseline (p = .001). No significant difference was observed at both the 6-month and 12-month follow-ups when CR was not received, even when lipid-modifying therapy, including statins, was initiated at baseline.

**Table 4:** Paired samples for LDL levels at 6 and 12 months follow up. ∗∗P < 0.01 and ∗P < 0.05 were considered significant. Lipid Drugs was lipid-modifying drugs including statins.

Groups:	N	Means LDL (mmol/L) at baseline	Means LDL (mmol/L) at follow up	Differences FU - Baseline	P-value
6 month follow up					
+ Lipid Drugs + CR	191	2.0 ± 0.91	1.7 ± 0.6	−.3	.001∗∗
+ Lipid Drugs - CR	17	2.0 ± 1.33	2.1 ± 1.3	.1	.821
- Lipid Drugs + CR	422	3.3 ± 1.03	1.7 ± 0.7	−1.6	.001∗∗
- Lipid Drugs - CR	13	2.9 ± 0.80	2.1 ± 1.3	−.4	.062
12 month follow up					
+ Lipid Drugs + CR	105	2.0 ± 0.8	1.7 ± 0.6	−.3	.000∗∗
+ Lipid Drugs - CR	13	1.9 ± 1.0	1.8 ± 0.7	**−**.1	.855
- Lipid Drugs + CR	195	3.3 ± 1.1	1.7 ± 0.8	−1.6	.000∗∗
- Lipid Drugs - CR	13	3.1 ± 0.9	2.5 ± 1.2	−.6	.156

Figure b from the supplementary material illustrates a decrease in LDL levels at 6-months and 12 months-follow-up in patients not receiving lipid-modifying drugs including statins at baseline, with the CR group showing the lowest levels. Figure c from the supplementary material illustrates a slight decrease in LDL levels at 6-month follow-up and similar results at 12-month follow-up.

### 3.5 Proportion of patients achieving LDL targets at 6 and 12 months follow-up

At both 6 and 12 months follow-up, a higher proportion of patients participating in CR achieved the LDL targets of <1.4 mmol/L and <1.7 mmol/L compared to those who did not participate in CR, this can be seen in Table B in the supplementary materials.

Among patients not receiving lipid-modifying drugs including statins at baseline, 36 % of those receiving CR reached LDL <1.4 mmol/L at 6 months, compared to 22 % in the non-CR group. Similarly, 56 % in the CR group achieved LDL <1.7 mmol/L versus 39 % in the non-CR group. These trends persisted at 12 months, with 37 % vs. 21 % reaching LDL <1.4 mmol/L, and 57 % vs. 36 % achieving LDL <1.7 mmol/L in the CR and non-CR groups, respectively.

In patients receiving lipid-modifying drugs including statins at baseline, a similar but less pronounced pattern was observed. At 6 months, 32 % of patients receiving CR reached LDL <1.4 mmol/L compared to 26 % in those without CR, while 52 % versus 47 % achieved LDL <1.7 mmol/L, respectively. At 12 months, 30 % versus 33 % reached LDL <1.4 mmol/L, and 48 % versus 67 % achieved LDL <1.7 mmol/L in the CR and non-CR groups, respectively.

### 3.6 Mortality

**Fig. 2:**
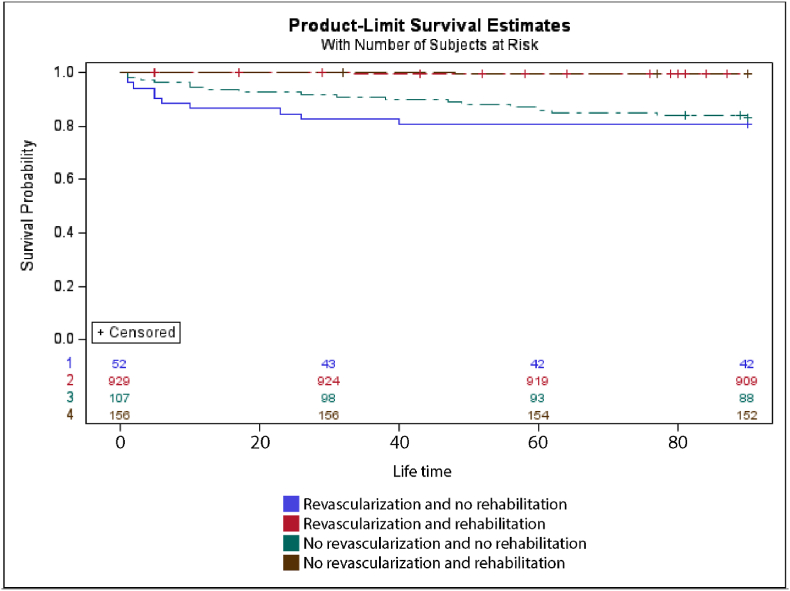
Kaplan-Meier survival carves comparing all-cause mortality according to revascularization status and participation in cardiac rehabilitation from discharge to 90 days. At 30 days, survival was highest in the 'Revascularization and rehabilitation’ group (approx. 97 %), followed by 'No revascularization + rehabilitation’ (96 %). The lowest survival was seen in the 'Revascularization and no rehabilitation' group (approx. 85 %).

Fig. 2 illustrates the mortality 90 days and 1 year after discharge in subjects who received revascularization. A statistically significant difference in mortality was seen 90 days after discharge when comparing the group who underwent revascularization and received CR and no CR (p = .0001). No significant difference was seen in one year follow-up, (p = .379).

Figure a in the supplementary illustrates the mortality of 90 days and 1 year after discharge in subjects who did not receive revascularization. A statistical difference in mortality was seen 90 days after discharge (p = 0.001) when comparing the groups who did receive CR and the group who did not. No significant difference was seen at one year-follow up in mortality (p = .132).

## 4 Discussion

In this study a total of 1209 MI survivors were included and 95.6 % of those participated in CR. A significant difference was seen in medical adherence in subjects who received CR, this was seen in both patients who underwent revascularization and those who were not revascularized. At both 6 and 12 months, there was a significant difference in LDL levels between the two groups: those receiving lipid-modifying drugs, including statins at baseline, alongside CR and those undergoing CR alone without lipid-modifying drugs at baseline.

### 4.1 Medication compliance

The adherence to guideline medication was associated with a significant difference between both revascularization and no revascularization when comparing CR with no CR at three months follow-up. However, a decline in adherence was observed at one year follow-up compared to three months follow-up. The results indicate that patients receiving CR are associated with an increase in adherence to guideline medication compared to not receiving CR. Our study findings are in line with the study by *Soldati* et al. [22] who found a strong association between CR participation and medication adherence observed among AMI patients who did not undergo percutaneous coronary intervention, at both 6 and 12 month follow up.

Our study findings are in line with the results from the study by *Thomson* et al. [23] suggested that CR increased the probability of being fully adherent to medication. Also, being fully adherent to medication upon entry to a CR program increases the odds of staying adherent at 6 months follow up [23]. This further emphasizes the importance of patient selection and the impact it could have on the findings in this study. The findings in this study could indicate that CR potentially has a positive impact on medication adherence. On the other hand, patients initiating CR may be associated with factors enhancing medical adherence.

While these findings demonstrate the positive effect of CR, it is important to notice that in both the revascularization and no revascularization groups, the number of patients not participating in CR was smaller compared to those who did. Non-participants in CR tended to be older, had a higher prevalence of diabetes, and were generally frailer. These characteristics likely contributed to their lower referral or participation rates in CR and may have influenced their overall clinical outcomes.

Such factors could influence not only the likelihood of participating in CR but also reflect a sicker, less engaged patient population. In contrast, those who participated in CR were likely healthier, had stronger social networks, were more motivated and had better access to healthcare resources. This potential selection bias could partially explain the positive outcomes observed in the CR group.

### 4.2 LDL level

The LDL level showed a statistically significant difference between 6 months and 12 months follow-up in all the groups who received CR. This was seen both when receiving lipid-modifying drugs including statins at baseline or not. No significant difference was observed at both the 6-month and 12-month follow-ups when CR was not received, even if lipid-modifying therapy, including statins, was initiated at baseline. Furthermore, LDL levels were consistently lower in the CR group compared to the non-CR group, suggesting that improved medical adherence associated with participation in CR could contribute to the reduction in LDL levels. These findings further show the positive impact of CR on lipid profiles, as no significant LDL changes were observed when patients did not receive CR. Additionally, in this study participation in CR was associated with a higher likelihood of achieving LDL targets levels after guidelines, particularly among patients not receiving statin or other lipid modifying drugs at baseline.

A study by *Wu* et al. [24] concluded that CR reduced low-density lipoprotein cholesterol (LDL-C) levels, triglyceride (TG) levels and total cholesterol (TC) levels and increased high-density lipoprotein cholesterol (HDL-C) levels [24]. These findings suggest that CR could have an impact on LDL levels, agreeing with the findings of this study, although they also conclude that more trials should be conducted for long-term CR [24]. CR could also result in a decrease in LDL through diet and exercise, although the focus on diet and exercise through CR differs from country to country.

### 4.3 Mortality

A significant difference in mortality was seen 90 days after discharge when comparing the group who received CR with the group who did not receive CR both in patients who underwent revascularization and who did not. Although, no statistical significance was seen from three months to one year follow up indicating that the first three months of discharge are the most impactful in mortality. Other studies have found similar results in mortality rates suggesting that receiving CR could impact the mortality [11,25].

### 4.4 Study limitations

This study is an observational study therefore confounding and exclusion of missing data could potentially lead to the results and be a limitation. Another limitation is that the survival analysis did not account for baseline characteristics, which could potentially influence the mortality rate. One limitation of this study that could influence the findings is patient selection. Patients undergoing CR may have a larger social network, higher levels of education, and, as our results show, are less frail compared to those who did not undergo CR. These factors could contribute to the significant differences observed in the study, potentially attributing them to factors beyond CR itself.

A limitation of this study is that we did not include other clinical events, such as revascularization rates or hospitalization rates, which are relevant outcomes in the context of CR. The primary focus was on CR participation, LDL control, medication adherence, and mortality. Future research incorporating these clinical events would provide a more comprehensive understanding of the broader impact of CR on patient outcomes. Another limitation of this study is that while 95 % of revascularized patients and 59 % of non-revascularized patients participated in a CR program, we did not assess completion rates of the CR programs. Whether patients who initiated CR also completed the program remain unknown and warrants further study.

## 5 Conclusion

In this study we showed that 95.6 % of the patients who underwent revascularization also participated in a CR program while this was 63.5 % among patients who did not undergo revascularization. The medical adherence for antithrombotic and lipid-modifying drugs including statin was increased in patients who participated in CR program both at three months and one year follow-up. The LDL levels were significantly lower in the patients who participated in the CR program. When looking at the mortality rate, a significant difference was seen at three month-follow up between patients who received CR program when compared to patients who did not undergo CR, however no significant difference in mortality rate was seen at one year follow-up. Our findings contribute to the understanding of CR program being an important step for the overall rehabilitation of patients with MI.

## CRediT authorship contribution statement

**Ahmad Agam:** Writing – review & editing, Writing – original draft, Methodology, Investigation, Data curation, Conceptualization. **David Vadsholt:** Methodology, Data curation. **Kristian Kragholm:** Writing – review & editing, Project administration, Methodology. **Lauge Klement Moltke Østergaard:** Writing – review & editing, Methodology. **Peter Bisgaard Stæhr:** Writing – review & editing. **Gitte Nielsen:** Writing – review & editing, Methodology, Funding acquisition. **Henrik Vadmann:** Writing – review & editing, Supervision, Methodology, Investigation.

## Data availability statement

Data are stored on secure servers on Statistics Denmark and cannot be shared according to Statistics Denmark regulations. Access to Statistics Denmark servers and the associated data can be granted by Statistics Denmark upon adequate permission.

## Funding information

Department funding only.

## Declaration of competing interest

The authors declare that there is no conflict of interest regarding the publication of this paper.
