# Supplementary material for: Association between cardiac rehabilitation and LDL-levels, adherence to guideline-recommended medication and mortality rate after myocardial infarction
Source: Int J Cardiol Cardiovasc Risk Prev. 2025 Jun 7;26:200444. doi: 10.1016/j.ijcrp.2025.200444 (PMC12182385; doi:10.1016/j.ijcrp.2025.200444)
Supplement: Multimedia component 1 [file mmc1.docx]

**Supplementary**

| Table A: Description of the cardiac rehabilitation program in Denmark (20) |
| --- |
| Physical training |
| Patient information and education |
| Psychosocial intervention, including job retention |
| Support for dietary changes |
| Support for smoking cessation |
| Optimization of pharmacological treatment |
| Clinical follow-up and maintenance of goals |

| Figure a: Mortaility rate one year follow up |
| --- |
| 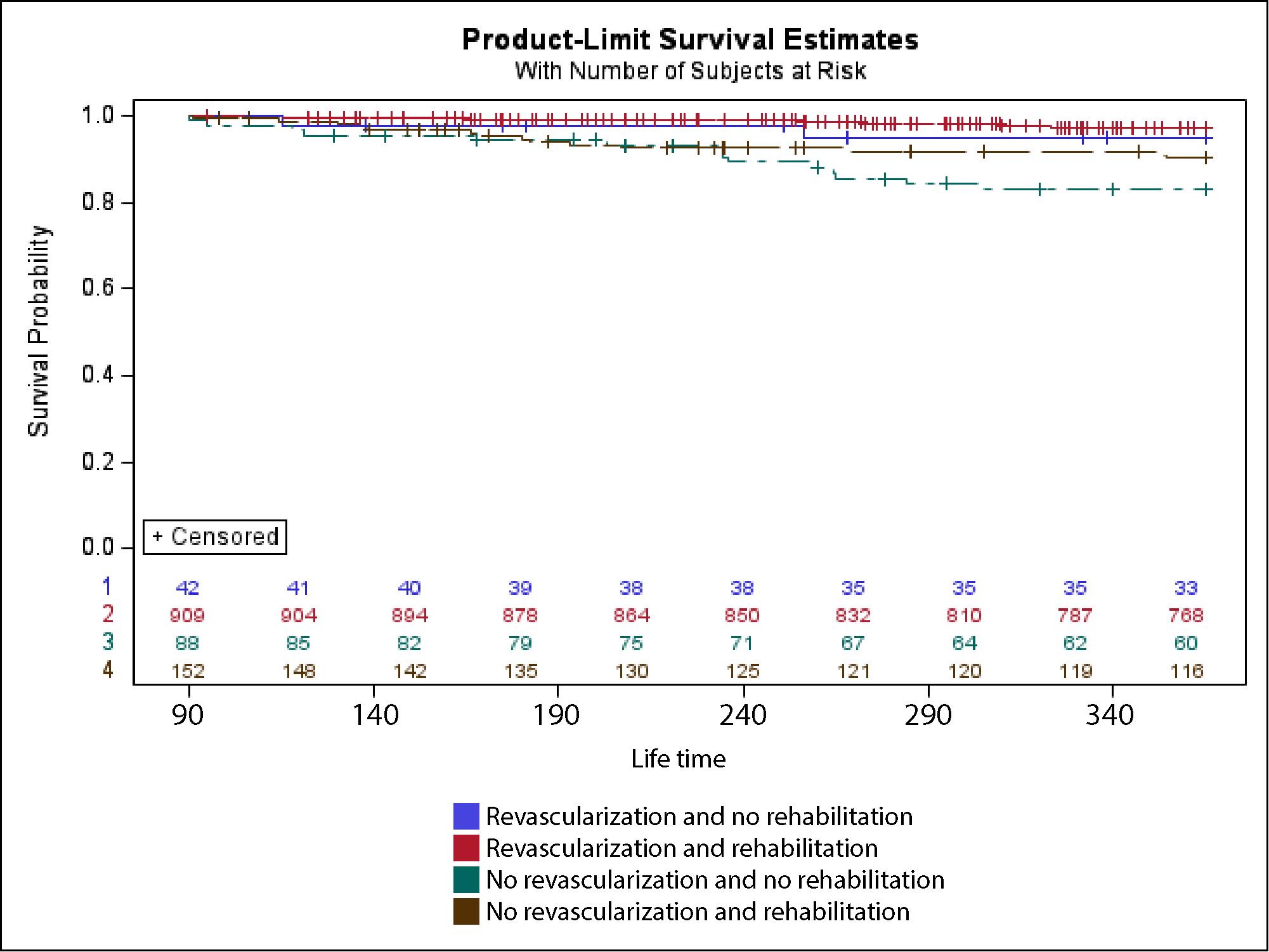 |

**Figure a:** Mortality rate from 90 days after discharge to one year follow-up after discharge in patients who did not receive revascularization.

| 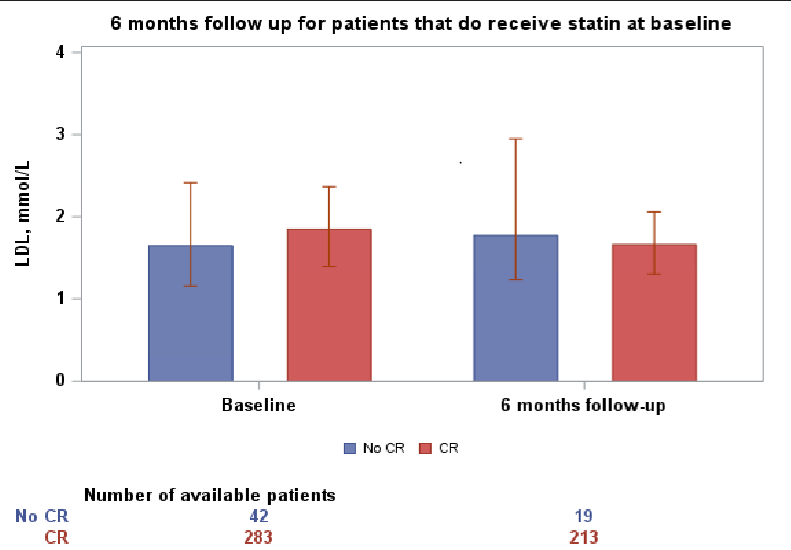 | 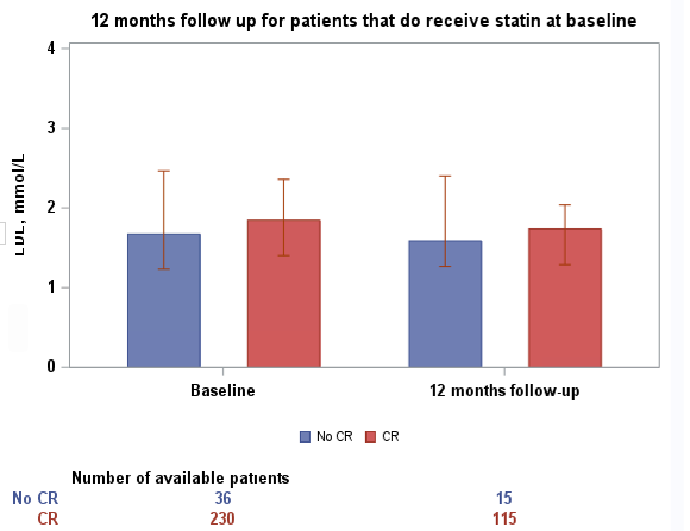 |
| --- | --- |

**Figure b:** Histogram of 6 months and 12 months follow up for patients that received statin at baseline illustrated with median and IQR.

| 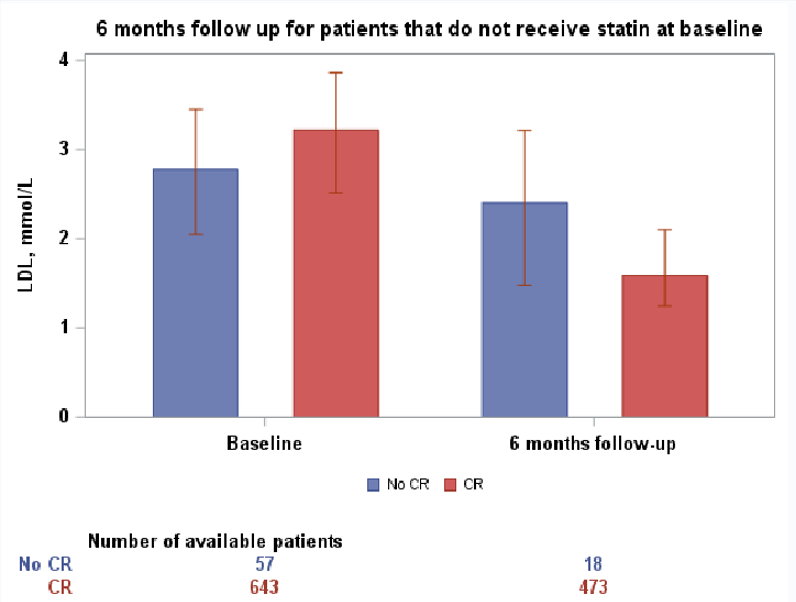 | 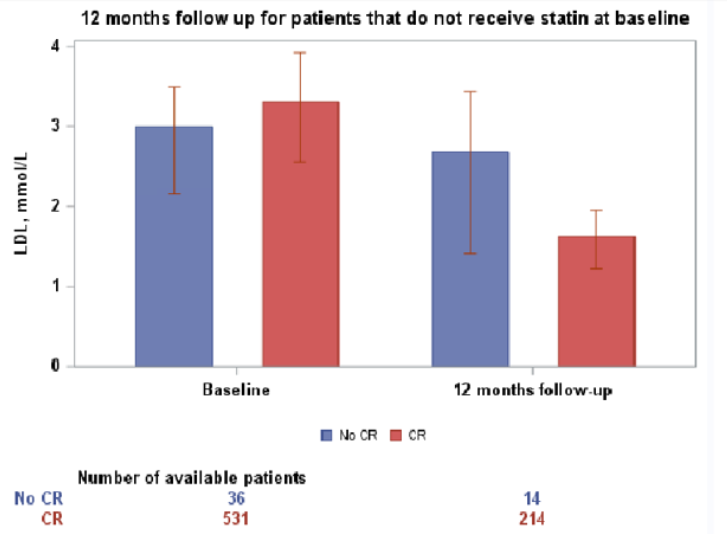 |
| --- | --- |

**Figure c:** Histogram of 6 and 12 months follow up for patients that do not receive statin at baseline illustrated with median and IQR.

| **Follow-up** | **Lipid drugs at baseline** | **CR-status** | **N** | **LDL < 1.4** mmol/L **(n)** | **%** | **LDL < 1.7** mmol/L **(n)** | **%** |
| --- | --- | --- | --- | --- | --- | --- | --- |
| 6 months | No | +CR | 473 | 171 | 36 | 266 | 56 |
| 6 months | No | -CR | 18 | 4 | 22 | 7 | 39 |
| 12 months | No | +CR | 214 | 79 | 37 | 122 | 57 |
| 12 months | No | -CR | 14 | 3 | 21 | 5 | 36 |
| 6 months | Yes | +CR | 213 | 67 | 32 | 111 | 52 |
| 6 months | Yes | -CR | 19 | 5 | 26 | 9 | 47 |
| 12 months | Yes | +CR | 115 | 35 | 30 | 55 | 48 |
| 12 months | Yes | -CR | 15 | 5 | 33 | 10 | 67 |

Table B. Proportion of patients achieving LDL targets at 6 and 12 months follow-up stratified by cardiac rehabilitation (CR) status and baseline statin or other lipid-modifying therapy.
